# The Effect of High-Frequency Repetitive Transcranial Magnetic Stimulation on Emotion Processing, Reappraisal, and Craving in Alcohol Use Disorder Patients and Healthy Controls: A Functional Magnetic Resonance Imaging Study

**DOI:** 10.3389/fpsyt.2019.00272

**Published:** 2019-05-07

**Authors:** Jochem M. Jansen, Odile A. van den Heuvel, Ysbrand D. van der Werf, Stella J. de Wit, Dick J. Veltman, Wim van den Brink, Anna E. Goudriaan

**Affiliations:** ^1^Department of Psychiatry, Amsterdam Institute for Addiction Research, Amsterdam UMC, University of Amsterdam, Amsterdam, Netherlands; ^2^Institute for Criminal Law and Criminology, Faculty of Law, Leiden University, Leiden, Netherlands; ^3^Department of Psychiatry, Amsterdam UMC, Vrije Universiteit Amsterdam, Amsterdam, Netherlands; ^4^Department of Anatomy and Neurosciences, Amsterdam Neuroscience, Amsterdam UMC, Vrije Universiteit Amsterdam, Amsterdam, Netherlands; ^5^Department of Research and Quality of Care, Arkin, Amsterdam, Netherlands

**Keywords:** alcohol use disorder, emotion reappraisal, craving, functional magnetic resonance imaging, emotion processing, repetetitive transcranial magnetic stimulation

## Abstract

Impaired cognitive–motivational functioning is present in many psychiatric disorders, including alcohol use disorder (AUD). Emotion regulation is a key intermediate factor, relating to the (cognitive) regulation of emotional and motivational states, such as in regulation of craving or negative emotions that may lead to relapse in alcohol use. These cognitive–motivational functions, including emotion regulation, are a target in cognitive behavioral therapy and may possibly be improved by neurostimulation techniques. The present between-subjects, single-blind study assesses the effects of sham-controlled high-frequency neuronavigated repetitive transcranial magnetic stimulation (10 Hz) of the right dorsolateral prefrontal cortex (dlPFC) on several aspects relevant for emotion regulation (emotion processing and reappraisal abilities) and related brain activity, as well as self-reported craving in a sample of alcohol use disorder patients (AUD; *n* = 39) and healthy controls (HC; *n* = 36). During the emotion reappraisal task, participants were instructed to either attend or reappraise their emotions related to the negative, positive, neutral, and alcohol-related images, after which they rated their experienced emotions. We found that repetitive transcranial magnetic stimulation (rTMS) reduces self-reported experienced emotions in response to positive and negative images in AUD patients, whereas experienced emotions were increased in response to neutral and positive images in HCs. In the functional magnetic resonance imaging (fMRI) analyses, we found that rTMS reduces right dlPFC activity during appraisal of affective images relative to sham stimulation only in AUD patients. We could not confirm our hypotheses regarding the effect of rTMS craving levels, or on reappraisal related brain function, since no significant effects of rTMS on craving or reappraisal related brain function were found. These findings imply that rTMS can reduce the emotional impact of images as reflected in blood oxygenation level-dependent (BOLD) response, especially in AUD patients. Future studies should replicate and expand the current study, for instance, by assessing the effect of multiple stimulation sessions on both explicit and implicit emotion regulation paradigms and craving, and assess the effect of rTMS within subgroups with specific addiction-relevant image preferences.

Clinical Trial Registration: www.ClinicalTrials.gov, identifier NCT02557815.

## Introduction

Harmful alcohol consumption ranks among the top five worldwide contributors of disease, disability, and death (1–3), and alcohol use disorder (AUD) is a common mental health disorder with a 12-month prevalence of 2–3% in the United States (4, 5) and 4% in Europe (6).

AUD is often described as a dual process disorder with reduced cognitive control and alterations in the brain reward system (7–9). Alterations in the reward circuitry include hypersensitivity to addiction relevant cues, in combination with hyposensitivity to natural rewards. This reward deficiency may lead to a disbalance in the reward system favoring addiction-relevant stimuli (10, 11). Brain alterations related to cognitive control include impaired (response) inhibition and emotion regulation (12), resulting in diminished ability to effectively control the emotional impact of certain thoughts or stimuli (13, 14). These changes in the reward circuitry and diminished control over emotions increase alcohol craving and relapse in remitted patients (15–17).

Emotion regulation can be described as the process of moderating the emotional impact of a thought or stimulus and may be achieved through various strategies ranging from relatively automatic and implicit (i.e., extinction) to explicit and cognitively controlled (reappraisal) (18). A recent review indicates that impaired emotion regulation is present in AUD based on various studies employing implicit (e.g., non-effortful) emotion regulation tasks such as emotion reactivity, implicit reappraisal, or behavioral control tasks (12). Impairments in reappraisal are supposed to be related to the development, persistence, and severity of substance dependence (19). Difficulties in coping with negative affect is one of the most prominent clinical factors in substance dependence (20), and the induction of negative affect may increase the urge to drink (16, 21), but studies on more controlled and explicit emotion regulation in substance use disordered patients are scarce. Explicit emotion reappraisal has been linked to several prefrontal brain areas: the dorsolateral prefrontal cortex (dlPFC) (for maintaining attentional and manipulating relevant information), ventrolateral prefrontal cortex (vlPFC) (for selecting the goal-appropriate interpretation), and dorsal anterior cingulate cortex (dACC) and dorsomedial prefrontal cortex (dmPFC) (both for conflict monitoring of the intended versus the actual behavioral outcomes) (18).

In our previous study within this special issue, we showed that *explicit* emotion regulation (reappraisal) abilities and related brain functioning were similar in alcohol use disorder (AUD) patients and healthy controls (HCs), but that in AUD patients compared to HCs, reduced brain activity during implicit emotion processing was present (Jansen et al., submitted). Based on these findings and the current literature, it seems that AUD patients are not impaired in explicit emotion regulation when actively instructed to apply these strategies, but that they do show reduced brain activity while watching emotional stimuli. A reduced response to non-addiction-relevant emotional cues in AUD patients may be related to a reduced salience of these non-addiction-related emotional stimuli.

Motivational interviewing and cognitive behavioral therapies are effective treatments for substance use disorders, including AUDs (22). Research suggests not only that psychological interventions should precede pharmacological treatment, but also that both types of treatment are effective (23, 24). After an initially successful period of abstinence, an estimated 50% of patients relapse into alcohol use within the first year (25–28). Similar results have been obtained for the pharmacotherapy of AUDs (25). These high relapse rates indicate that research into new treatment possibilities is warranted.

Noninvasive neurostimulation of the prefrontal cortex, using techniques such as repetitive transcranial magnetic stimulation (rTMS), may offer a new alternative intervention method for substance use disorder patients (29, 30). rTMS and other forms of neuromodulation can reduce acute craving in patients with a substance use disorder (31), especially when stimulating the (right) dlPFC (29) and improve cognitive functions such as attention, memory, and executive functioning in patients with substance use disorders (32, 33). In recent years, increased attention has been directed toward improving emotion processing and emotion reappraisal with prefrontal rTMS, often directed toward the dlPFC, which is central in explicitly controlled emotion regulation strategies, including reappraisal (18). These studies vary in their methodology and reveal contradictory results with high-frequency *right* dlPFC rTMS being associated with an increase in attentional bias toward negative stimuli (34), whereas high-frequency *left* dlPFC stimulation decreased the amygdala response to negative stimuli (35). Additionally, a recent review concludes that rTMS influences cognitive control and the attentional and affective aspects of emotion regulation and that rTMS should be investigated for substance use disordered patients (33, 36).

The five rTMS studies that are discussed by Choi et al. (36) use varying methodological approaches regarding stimulation location (right and/or left dlPFC, cerebellum), stimulation frequency (high and low frequency), and study outcome (autonomic reactions, attention, mood, and affective processing). De Raedt et al. (37) and Vanderhasselt et al. (34) investigated the effects of sham-controlled high-frequency rTMS of the dlPFC on attentional aspects of emotion regulation, and both conclude that right dlPFC rTMS increased attention toward—or decreased disengagement of—negative stimuli. One study employing sham-controlled high- and low-frequency stimulation of the right dlPFC shows that low-frequency (but not high-frequency) stimulation increased heart rate deceleration in response to negative and neutral, relative to positive, pictures (38). Another study employing low-frequency stimulation of the right dlPFC shows increased responses to fearful faces compared to neutral faces in the right temporal junction (39). Finally, Schutter and van Honk (40) showed that sham-controlled low-frequency stimulation of the cerebellum increased negative mood after an emotion regulation task. Additionally, more recent findings are mixed: high-frequency right dlPFC rTMS stimulation was found not to influence heart rate reactivity to positive or negative images (38) or emotion recognition performance (41). Notzon et al. (42), on the other hand, found high-frequency rTMS of the right DLPFC, compared to low-frequency rTMS, to improve emotion discrimination, leading the authors to conclude that high-frequency rTMS leads to better cognitive control over aversive stimuli. Despite the variety in applied study methods, these studies indicate that rTMS may influence emotion processing and reappraisal in healthy subjects. Other studies suggest that the effect of rTMS may be different in persons with a psychiatric disorder (32), and in a recent study, we have shown that high-frequency *left* dlPFC stimulation may reduce self-reported affect related to negative images in obsessive–compulsive disorder patients and that it reduces dorsomedial prefrontal cortex (PFC) activity relative to sham stimulation, independent of task conditions (43). This study is one of the few studies to investigate the effect of rTMS on both emotion processing and reappraisal at a behavioral and neural level. There are currently no rTMS studies on emotion processing and reappraisal in AUD patients, while these processes are highly relevant for the treatment of this disorder. Cognitive behavioral therapies, for example, often include some form of emotion regulation training (44, 45).

The current study is the first to investigate the effect of high-frequency rTMS on emotion processing and reappraisal in AUD patients and HCs at a behavioral and neural level. Based on previous studies (29), we hypothesize that high-frequency rTMS of the right dlPFC ameliorates reappraisal and the recruitment of the reappraisal-related brain network in both AUD patients and HCs, but this improvement is expected to be greater in the AUD group compared to the HC group [see Ref. (32)]. We expect that high-frequency stimulation will influence (increase or decrease) emotion processing at a behavioral and neural level. Finally, we expect that in AUD patients, high-frequency rTMS decreases reappraisal task-induced craving.

## Methods

This study is part of a larger study, with two fMRI sessions, focusing on differences in emotion regulation performance and related brain activity between AUD patients and HCs during the first (baseline) session and the effect of rTMS on craving, emotion regulation, and related brain activity during the second (rTMS stimulation) session. For a description of the main task effects (e.g., experimental manipulation during the first session), as well as the between-participant group differences at baseline (ADP vs. HC), please see our previous manuscript within this special issue (Jansen et al., submitted). The current manuscript describes the effects of rTMS on emotion processing, reappraisal, craving, and related brain activity.

### Participants

A total of 39 AUD patients (26 males) and 36 HCs (20 males) were included in this between-subjects study and were matched on (mean) age, sex, and education. AUD patients were sober for at least 3 weeks and were recruited from addiction treatment centers in the larger city area of Amsterdam, the Netherlands. Sobriety was confirmed with a urine test in the research lab on the test days. None of the participants used psychoactive medication, cannabis, opioids, or stimulants. HCs were recruited through Internet and social media advertisements. All participants were screened for MRI suitability. All subjects were screened (and if positive excluded) for the presence or a history of psychiatric disorders, including substance abuse or dependence, using the Composite International Diagnostic Interview (CIDI) (46). The study was approved by the local Medical Ethical Commission of the Academic Medical Center of the University of Amsterdam and participants signed the informed consent form, consistent with the Declaration of Helsinki, before participating in the study. Participants were remunerated for their participation.

### Questionnaires

In addition to the CIDI interview, the Alcohol Use Disorder Identification Test (AUDIT) (47), Beck’s Depression Inventory (BDI) (48), Beck’s Anxiety Inventory (BAI) (49), the Toronto Alexithymia Scale-20 (TAS-20) (50), and the Emotion Regulation Questionnaire (ERQ) (51) were administered to assess alcohol problem severity, depression severity, anxiety severity, alexithymia, and emotion regulation, respectively. Finally, craving was assessed with the Alcohol Urge Questionnaire (AUQ) (52) before and after the performance of the emotion reappraisal task in both sessions.

### Emotion Reappraisal Task

Two matched versions of the task were programmed in E-Prime 2.0 and presented in a counterbalanced order in two different sessions during fMRI scanning. Each session, participants viewed nine negative, nine positive, nine neutral, and nine alcohol-related images on a screen using a mirror attached to the head coil. The negative, positive, and neutral images used in this task were selected from the International Affective Picture Set (IAPS) (53). Negative images had a low valence (≤4.0) and high arousal (≥6.0), neutral images had a mildly positive valence (4.5 < *x* < 7.0) and low arousal (2.0 < *x* < 4.2), and positive images had high valence (≥7.0) and high arousal (≥5.0), based on the original IAPS scores. The alcohol-related images were selected from Vollstädt-Klein et al. (54) and supplemented by alcohol-related images of popular Dutch alcoholic beverages. All alcohol-related images were separately validated in an independent sample of both HCs and AUD patients (*n* = 17) for valence (mildly positive: 3.0 < *x* < 6.0) and arousal (low: 2.0 < *x* < 4.0).

The images were paired with one of two different instructions: “attend” and “reappraise.” In the attend instruction, participants were told to view and identify themselves with the situation in the image (e.g., “how would you feel in this situation”). In the reappraise condition, participants were told to reappraise their emotions related to these images in such a way that the emotional significance was reduced (e.g., “imagine a less negative outcome or interpretation”). Images were presented in 12 blocks of three images of the same emotion type (negative, positive, neutral, and alcohol) with the same instruction (attend and reappraise) and presented in a pseudo-randomized order.

After each image, for both instructions (attend and reappraise), a visual analogue scale (VAS) was presented. Participants had to rate their emotional state (“How do you feel?”) by moving a bar to the right or left by pressing a button box multiple times. A moving bar was set in the middle of a line (representing a neutral value of 50) and the range of emotions on this line was indicated by previously validated self-assessment manikins depicting valence (55). Indicated values ranged from 0 (negative, extreme left of the line) to 100 (positive, extreme right of the line). Prior to scanning, the assessment was explained and practiced outside the scanner using example stimuli (not used in the experiments) for approximately 5 min (for more information, see **S1**). The reappraisal task itself took approximately 25 min.

### Repetitive Transcranial Magnetic Stimulation

In the stimulation session, participants received either (single-blind) neuro-navigated (Visor2, ANT) sham or active right dlPFC rTMS using a MagStim Rapid2 Air-film coil with a 70-mm diameter (MagStim Co., UK) immediately before entering the MRI. The active rTMS consisted of sixty 5-s trains of 10 Hz at 110% motor threshold (31). These parameters are within the international safety limits for use of rTMS (56). The stimulation location was defined for each individual separately as the most significant peak voxel in the right dlPFC activated during the reappraisal task in the baseline session for the [reappraise minus attend] contrast, as defined by the BrainMap database (57). Sham stimulation was performed using identical parameters, but the rTMS coil was tilted 90° relative to the skull (58).

### Analysis

#### Behavioral Analysis

Data were prepared for analysis by winsorizing extreme values for experienced emotion (mean VAS per condition and session) and craving (AUQ pre- and post-scores), by replacing values below the 5th and above the 95th percentile with the 5th or 95th percentile, respectively, and by confirming that experienced emotion was normally distributed.

In order to assess effects of stimulation (rTMS/sham), image type (positive/neutral/negative/alcohol), instruction (attend/reappraise), and participant group (AUD/HC) on experienced emotion, a four-way general linear model (GLM) Univariate ANOVA was performed, including experienced emotion after rTMS (condition-specific mean VAS) as the dependent variable, and instruction, image type, participant group, and stimulation as fixed factors. Condition-specific experienced emotion during the first session (before rTMS) was incorporated as a covariate. Significant interactions were followed up by Bonferroni-corrected simple effects analyses.

The AUQ was administered before (pre) and after (post) the reappraisal task during each session. Due to the many mistakes that were made in the second and seventh AUQ question—which are reverse coded and were misinterpreted—these were excluded from the analysis. Pre- and post-scores on both sessions were positively skewed and therefore a log(*x* + 1) transformation was applied. A GLM Univariate ANOVA was performed including AUQ scores as the dependent variable, time (pre/post) as the within-subjects factor, and both stimulation (rTMS/sham) and participant group (AUD/HC) as the between-group factor.

#### Functional Magnetic Resonance Imaging

##### Data Acquisition

MRI scanning was performed on a Philips Achieva 3T scanner at the Spinoza Imaging Centre, Amsterdam, the Netherlands. Functional MRI [echo time (TE) = 27.63 ms; repetition time (TR) = 2,000 ms; field of view (FOV) = 240 × 240 mm, 37 3-mm slices, 0.3-mm slice gap; 80 × 80 matrix; flip angle = 76.1°] was performed to acquire blood oxygenation level-dependent (BOLD) signals using single-shot multi-echo (59) T2*-weighted echo planar imaging (EPI). These T2-weighted flow-compensated eight spin-echo anatomical images were oriented axially along the anterior commissure to the posterior commissure (AC–PC) line. During the baseline session, a T1-weighted 3D data set was obtained for anatomical reference; TR = 8.196 ms, TE = 3.73 ms, field of view (FOV) = 140 × 188 × 220 mm, 240 × 187 matrix, flip angle = 8°, slice thickness = 1 mm, number of slices = 220.

##### Pre-Processing and First-Level Analysis

Pre-processing was performed with SPM8 (Wellcome Trust Centre for Neuroimaging, London, United Kingdom) in MATLAB (version 2012b) and included realignment to the first image, slice timing correction to the middle (18th) slice, coregistration of the anatomical T1 of the subject to the mean functional scan, and warping of this coregistered T1 to standard space. Next, the volumes were normalized to the Montreal Neurological Institute (MNI) template and smoothed with a 7-mm Gaussian kernel in order to increase signal-to-noise ratio. To account for low-frequency drifts, a high-pass filter (128 Hz) was applied. Three subjects (2 AUD, 1 HC) were removed due to low quality of the fMRI data (e.g., scanner artifacts).

In the first-level model, regressors of no interest were instruction and VAS scoring. Instruction was modeled with boxcars of 3 s, whereas VAS scoring was modeled with a boxcar for the true duration of the scoring process since this was self-paced. The eight regressors of interest included the onsets of the negative, positive, neutral, and alcohol-related images in either attending or reappraising condition, which were modeled as boxcars (duration, 5 s) and convolved with a hemodynamic response function, in the first-level, single-subject, fixed-effects analysis. First-level contrasts for reappraisal [reappraise > attend] were computed per emotion condition (negative, positive, alcohol, and neutral). For emotion processing, separate contrasts were created for attending emotional images (alcohol, positive, or negative) versus neutral images [attend emotion (positive, negative, alcohol) > attend neutral].

##### Functional Magnetic Resonance Imaging Data Analysis

In order to assess the effects of rTMS on emotion processing and emotion reappraisal, separate second-level fMRI analyses were performed.

For the attend condition (emotion processing), a 3 × 2 × 2 ANOVA was conducted in SPM12, including the [attend emotion > attend neutral] contrast per image type (alcohol, positive, and negative), in order to assess the interaction between image type (alcohol, positive, and negative), group (AUD and HC), and stimulation (rTMS and sham). Additionally, two-way interactions (group by stimulation, image type by stimulation, and group by emotion type) were assessed.

For the reappraise condition, a 4 × 2 × 2 ANOVA was conducted in SPM12, including the [reappraise > attend] contrasts per image type, in order to assess the interaction between image type (alcohol, neutral, positive, and negative), group (AUD and HC), and stimulation (rTMS and sham). Additionally, two-way interactions (group by stimulation, image type by stimulation, and group by image type) were assessed. All results are reported at a whole brain *p* < 0.05 family wise error (FWE)-corrected threshold.

## Results

### Demographics

AUD patients and HCs were successfully matched on age, gender, and years of education. However, AUD patients reported significantly higher levels of smoking, depression (BDI), anxiety (BAI), and alexithymia (TAS-20). Analyses were not corrected for these differences, because depression, anxiety, and alexithymia levels are well known to be elevated in AUD (60–62) and are related to emotion processing and reappraisal, and thus correction for these factors could results in false-negative findings. Remarkably, there were no group differences in the ERQ scores (**Table 1**). Furthermore, there were no significant differences on any of the questionnaires between participants receiving active rTMS or sham rTMS.

**Table 1:** Sample characteristics.

	Mean AUD (sd) *n* = 39	Mean HC (sd) *n* = 36	Significance
Age	41.64 (8.63)	43.75 (10.90)	*t*(1,73) = .93, *p* = 0.35
Years of education	15.31 (3.05)	15.53 (2.85)	*t*(1,71) = .49, *p* = 0.62
Gender	M = 26	M = 20	χ^2^(1,73) = .97, *p* = 0.32
AUDIT	22.11 (10.51)	4.23 (2.52)	*t*(1,70) = 9.80, *p* < 0.001
Current smoker	Yes *n* = 29/35 (82.9%)	Yes *n* = 10/32 (31.3%)	χ^2^(1,67) = 18.30, *p* < 0.001
TAS-20 total	51.43 (10.83)	42.97 (8.88)	*t*(1,65) = 3.48, *p* = 0.001
TAS-20 DIDF	31.83 (8.16)	24.82 (7.40)	*t*(1,66) = 3.71, *p* < 0.001
TAS-20 EOT	11.97 (3.30)	11.27 (2.78)	*t*(1,69) = .98, *p* = 0.33
ERQ total	37.81 (7.95)	36.58 (8.42)	*t*(1,73) = .90, *p* = 0.37
ERQ Reappraisal	20.22 (5.87)	19.00 (7.53)	*t*(1,71) = .76, *p* = 0.45
ERQ Suppression	17.72 (5.01)	17.58 (5.08)	*t*(1,71) = .01, *p* = 0.99
Beck Depression Inventory	10.84 (9.58)	4.33 (6.36)	*t*(1,72) = 3.41, *p* = 0.001
Beck Anxiety Inventory	30.40 (8.73)	24.25 (4.67)	*t*(1,74) = 3.75, *p* < 0.001

AUD, alcohol use disorder; HC, healthy controls; SD, standard deviation; AUDIT, Alcohol Use Disorders Identification Test; TAS, Toronto Alexithymia Scale; DIDF, difficulties identifying and describing feelings; EOT, externally oriented thinking; ERQ, emotion regulation questionnaire. ERQ Reappraisal and Suppression are subscales of the ERQ.

This table shows the results for the analyses of the sample characteristics. Values are denoted as mean (standard deviation). Total number of participants per comparison may vary due to a small number of missing values.

### Repetitive Transcranial Magnetic Stimulation Effects

#### Emotion Processing and Reappraisal

The four-way repeated-measures ANOVA with experienced emotion (mean VAS per condition after rTMS) as the dependent variable, image type (negative, positive, neutral, and alcohol), instruction (attend and reappraise) as within-subject factors, and participant group (AUD and HC) and stimulation (rTMS and sham) as between-subject factors—while correcting for baseline experienced emotion (mean VAS per condition) before rTMS—did not reveal a significant four-way interaction [*F*(3,559) = .11, *p* = 0.95, *d* < 0.01]. There was, however, a significant three-way interaction between image type, participant group, and stimulation [*F*(3,559) = 7.18, *p* < 0.001, *d* = 0.39].

**Figure 1:**
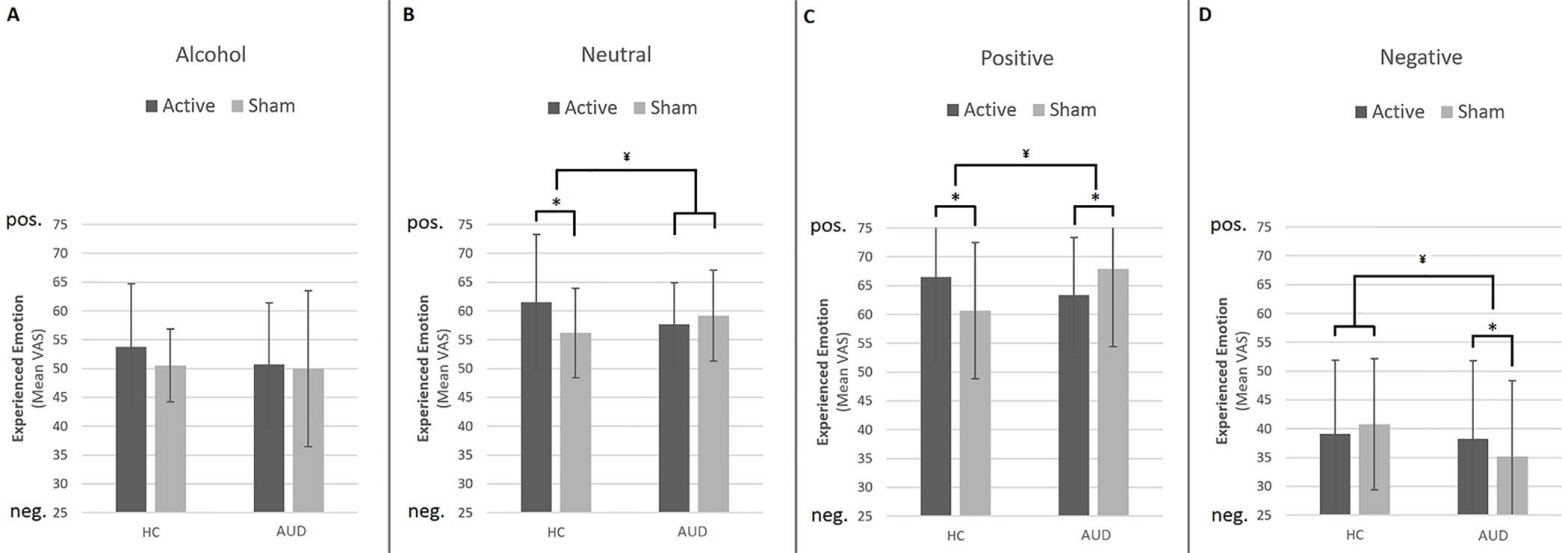
Effect of repetitive transcranial magnetic stimulation (rTMS) on experienced emotion. This figure shows the differential effects of rTMS and sham stimulation on experienced emotion in reaction to alcohol **(A)**, neutral **(B)**, positive **(C)**, and negative **(D)** images. Note that a value of 50 represents “neutral” experienced emotion. Bars represent estimated marginal means, which are corrected for experienced emotion before rTMS. Error bars are standard deviations from the mean. ¥ = significant two-way interaction (group by stimulation), * = significant main effect of stimulation within participant group, pos. = positive, neg. = negative.

To interpret the significant three-way interaction, we conducted separate GLM Univariate ANOVAs per image type (negative, positive, neutral, and alcohol related), including experienced emotion (mean VAS per condition after rTMS) as the dependent variable, and participant group (AUD and HC) as well as stimulation (rTMS and sham) as between-subject factors—while correcting for baseline experienced emotion (mean VAS per condition) before rTMS. The results of these analyses reveal significant interactions between participant group and stimulation for negative [*F*(1,143) = 4.86, *p* = 0.03, *d* = 0.37], positive [*F*(1,143) = 18.38, *p* < 0.001, *d* = 0.11], and neutral images [*F*(1,143) = 6.48, *p* = 0.01, *d* = 0.04], but not for alcohol-related images [*F*(1,143) = .12, *p* = 0.73, *d* = 0.06] (see **Figure 1**).

Simple effects analyses show that rTMS dampens experienced emotions in response to positive, neutral, and negative images in AUD patients, whereas in HCs, rTMS intensifies experienced emotions in response to positive and neutral images. For example, experienced emotion in reaction to positive images is more positive after rTMS [mean (*m*) = 66.50, standard deviation (sd) = 14.66] compared to sham (*m* = 60.66, sd = 11.82) stimulation in HCs [*F*(1,69) = 7.37, *p* = 0.008, *d* = 0.65], whereas in AUD patients [*F*(1,73) = 7.07, *p* = 0.01, *d* = 0.62], experienced emotion to these images is less positive (e.g., more neutral) after rTMS (*m* = 63.39, sd = 9.99) compared to sham stimulation (*m* = 67.87, sd = 13.46). The simple effects analyses for neutral and negative images reveal that rTMS (*m* = 61.52, sd = 11.77) significantly increases positive experienced emotions to neutral images relative to sham stimulation (*m* = 56.18, sd = 7.72) in HCs [*F*(1,69) = 5.98, *p* = 0.02, *d* = 0.59], but not in AUD patients [*F*(1,73) = 1.00, *p* = 0.32, *d* = 0.24]. Finally, rTMS (*m* = 38.26, sd = 13.52) dampens negative emotions in response to negative images in AUD patients relative to sham stimulation [*m* = 35.18, sd = 13.13; *F*(1,73) = 7.07, *p* = 0.01, *d* = 0.62], but does not affect experienced emotion in HCs [*F*(1,69) = .06, *p* = 0.81, *d* = 0.06].

#### Craving

**Figure 2:**
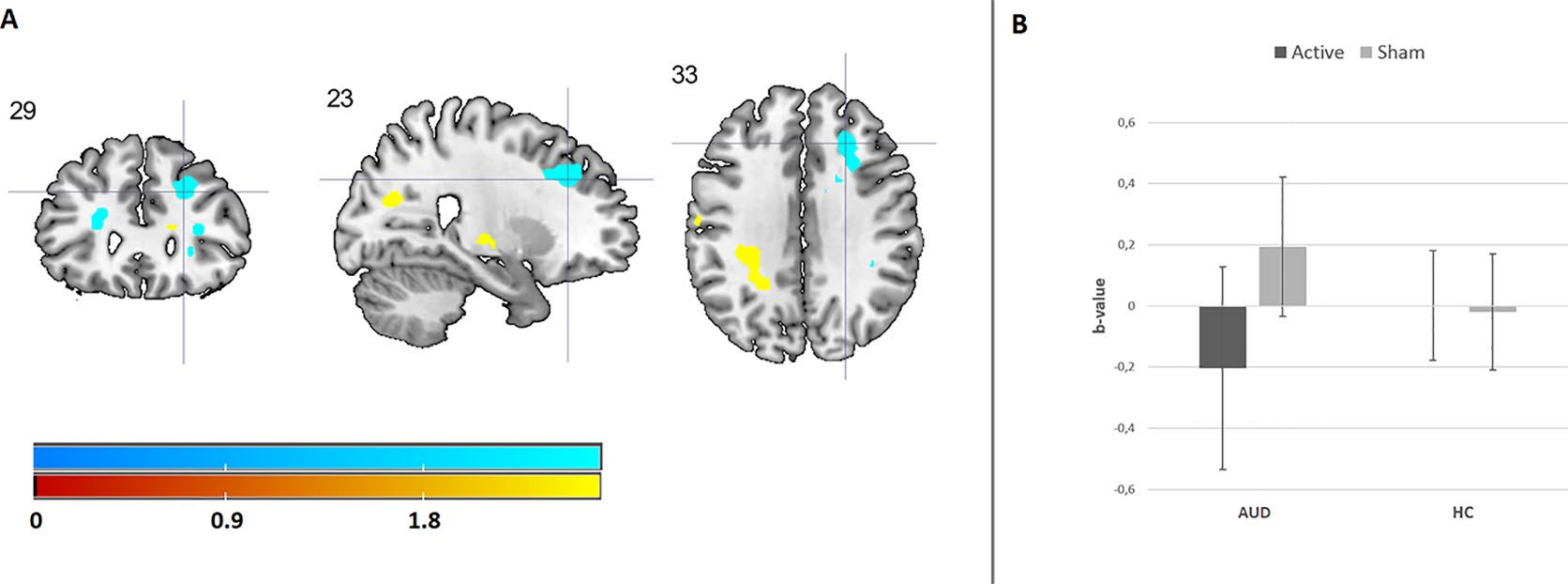
This figure shows the trend-significant interaction between emotion (alcohol, neutral, positive, negative) and stimulation (rTMS, sham) within the superior frontal gyrus. **(A)** This panel shows the location for the interaction in the bilateral superior frontal gyrus. For illustrative purposes, these results are depicted at a p < 0.001 uncorrected threshold **(B)**. This panel shows the interaction within the peak voxel in the right superior frontal gyrus, based on the extracted beta weights.

The results from the GLM univariate ANOVA with craving levels as the dependent variable, time (pre and post) as within-subjects factor, participant group (AUD and HC), and stimulation (rTMS and sham) as between-subjects factors did *not* reveal a three-way interaction [*F*(1,132) = 1.70, *p* = 0.20, *d* = 0.23]. There was, however, a significant two-way interaction between group and stimulation [*F*(1,132) = 4.64, *p* = 0.03, *d* = 0.38], but not between group and time [*F*(1,132) = .36, *p* = 0.55, *d* = 0.11] or between time and stimulation [*F*(1,132) = .90, *p* = 0.34, *d* = 0.17]. These results indicate that stimulation (rTMS and sham) did not differentially affect the change in craving over time (pre and post) for AUD patients and/or HCs, and therefore do not support our hypothesis that rTMS would reduce craving levels relative to sham stimulation (see **Figure 2**).

### Functional Magnetic Resonance Imaging Results

#### Emotion Processing

**Figure 3:**
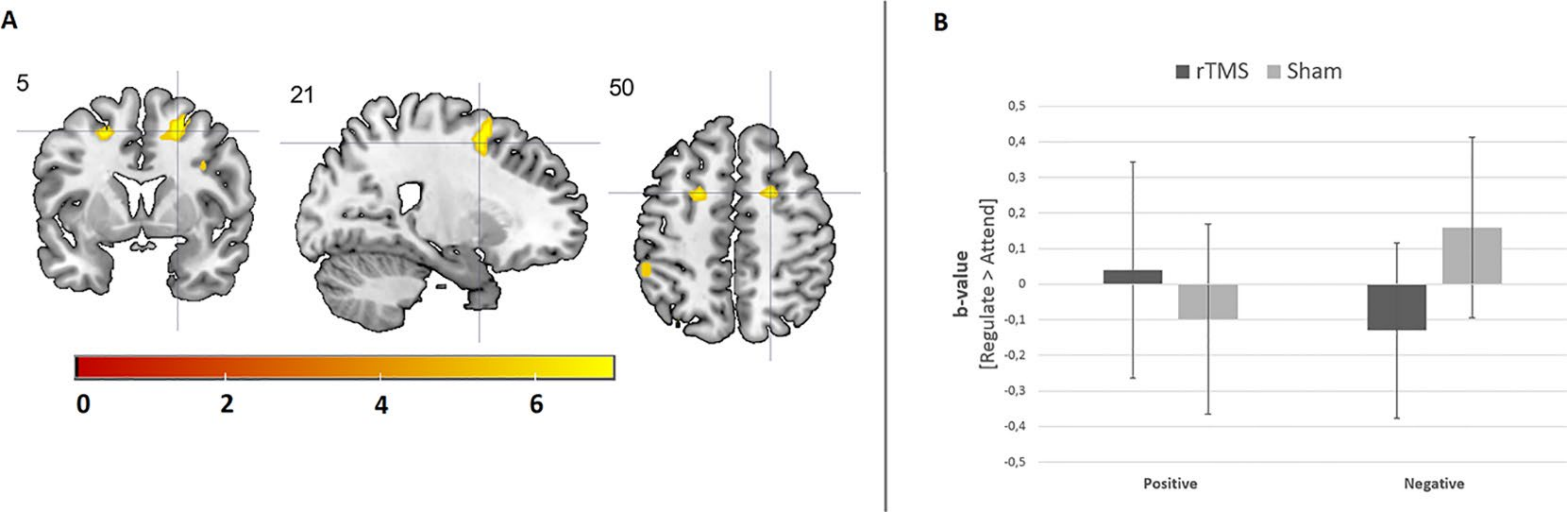
The effect of rTMS on brain activity during emotion processing. **(A)** Cool coloring represents brain activity in the dorsolateral prefrontal cortex (dlPFC), which has decreased due to rTMS relative to sham stimulation in AUD patients. Hot coloring indicates brain activity in the supramarginal gyrus, which has increased due to rTMS stimulation relative to sham stimulation in AUD patients. For illustrative purposes, these results are depicted at a p < 0.001 uncorrected threshold. **(B)** This bar chart shows the effect of rTMS and sham stimulation on right dlPFC activity in AUD patients and HCs.

Results from the 3 × 2 × 2 ANOVA, including the [attend emotion > attend neutral] contrast per image type, did not reveal a three-way interaction between image type (alcohol, positive, and negative), group (AUD and HC), and stimulation (rTMS and sham). The results do show a significant two-way interaction between group and stimulation within the right dlPFC (see **Figure 3** and **Supplementary Information**), originating from a decrease in dlPFC brain activity after rTMS relative to sham stimulation in the AUD group. The other two-way interactions (emotion by stimulation and group by image type) did not reveal any significant effects.

#### Emotion Reappraisal

**Figure 4:**
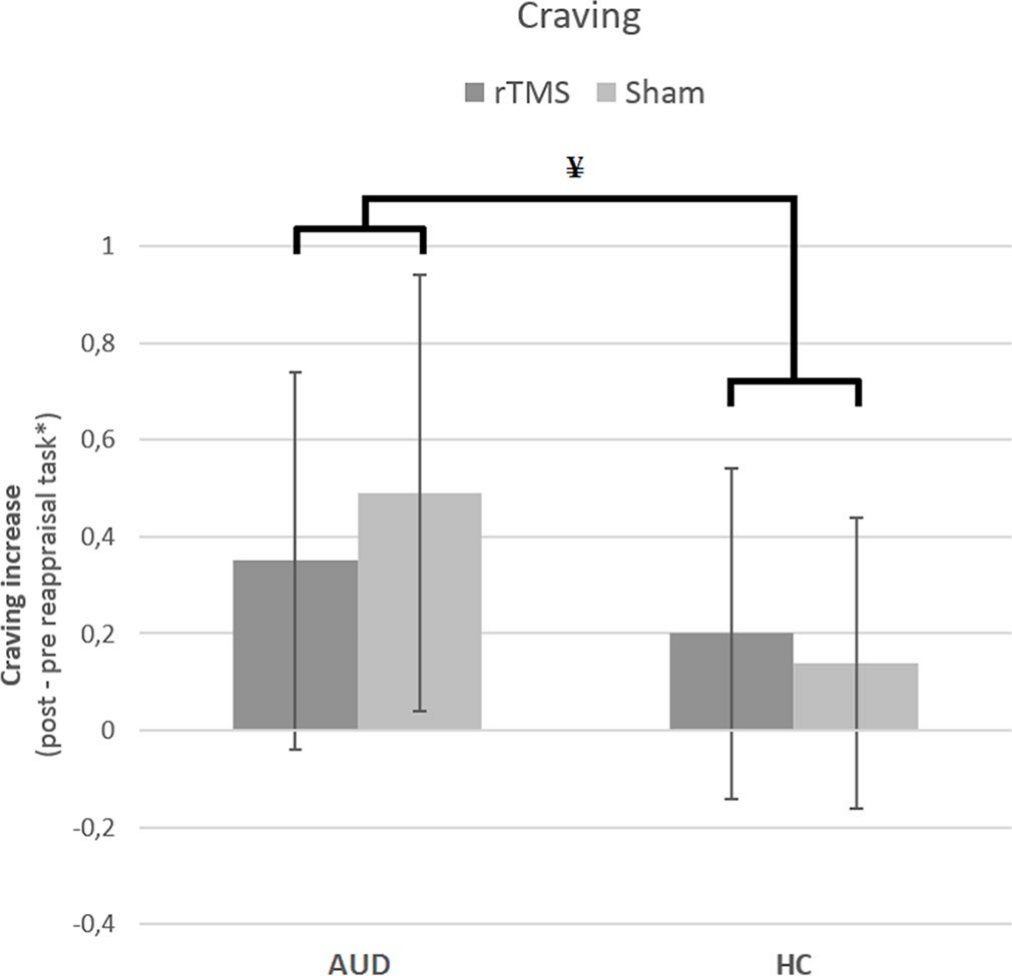
The effect of rTMS and sham stimulation on craving per group. This figure depicts the significant interaction between stimulation (rTMS, sham) and group [alcohol use disorder (AUD), healthy controls (HC)]. * Note that the values are log(x + 1) transformed. ¥ = significant two-way interaction.

Results from the 4 × 2 × 2 ANOVA, including the [regulate > attend] contrast per emotion, did not reveal a three-way interaction between image type (alcohol, neutral, positive, and negative), group (AUD and HC), and stimulation (rTMS and sham). Although none of the two-way interactions reached significance, there was a trend-significant interaction between image type (alcohol, neutral, positive, and negative) and stimulation (rTMS and sham; *p* < 0.1, FWE corrected). Follow-up analyses revealed that this two-way interaction originated from a difference in the effect of rTMS on brain activity between the reappraisal of positive and negative images in the bilateral superior frontal gyrus for both AUD patients and HCs. rTMS stimulation decreased superior frontal gyrus activity in response to negative images relative to sham stimulation, whereas rTMS increased activity in this area in response to positive images (see **Figure 4** and **Supplementary Information**).

## Discussion

The purpose of this study was to investigate the effect of sham-controlled high-frequency right dlPFC rTMS on emotion processing, reappraisal ability, and related brain functioning in alcohol use disorder patients (AUD patients) and healthy controls (HCs). We hypothesized that stimulation of the right dlPFC would improve emotion processing and reappraisal—especially in AUD patients—and alter the recruitment of the reappraisal-related brain network. In line with our hypotheses, we found that rTMS reduces self-reported experienced emotions in response to positive and negative images in AUD patients, whereas experienced emotions were increased in response to neutral and positive images in HCs. Instruction (attend or reappraise) did not influence these results. In the fMRI analyses, we found that rTMS reduces right dlPFC activity during appraisal of affective images relative to sham stimulation only in AUD patients. Our results do not support our hypothesis regarding the effect of rTMS on reappraisal-related brain function, since no significant effects of rTMS on reappraisal-related brain function were found. On a lower significance level, however, rTMS—compared to sham stimulation—*decreased* activity during reappraisal of negative images and *increased* activity in the bilateral superior frontal gyrus during reappraisal of positive images in both AUD patients and HCs. rTMS did not influence the change in craving levels compared to sham stimulation.

At a behavioral level, rTMS stimulation reduced the impact of affective (neutral, positive, and negative) images in AUD patients, but increased the impact of positive and neutral images in HCs. No effect of rTMS was found for alcohol-related images in either group, which is not in line with our hypotheses. AUD is characterized by reduced salience of natural stimuli relative to addiction-relevant cues (11), but alcohol consumption has also been suggested as a self-medication strategy to reduce—relapse-related—stress and negative emotions (63, 64). Therefore, reducing the impact of emotional images in AUD patients through rTMS may be related to reduced emotional impact of affective stimuli, which could possibly reduce stress, craving, and subsequent relapse. This explanation is supported by previous studies, which show positive effects of rTMS on craving reduction (31), cognitive functioning (32, 33), and depressive symptoms (65).

Our results furthermore suggest that rTMS stimulation affects emotion processing and reappraisal in HCs and AUD patients differently, since rTMS reduced emotional experience in AUD patients and reduced right dlPFC activity during emotion processing, whereas experienced emotion was increased in HCs and no effect was found on related brain activity. Although, to our knowledge, there are no other studies on the effect of rTMS on emotion processing and reappraisal in AUD patients, these results are in line with a review that suggests that rTMS effects may differ between healthy and patient populations (32).

These results correspond with previous studies in HCs that reveal that rTMS influences emotion processing (34, 35) and reappraisal (35, 42) in HCs. rTMS increased attentional bias toward negative stimuli in a study in HCs (34) and lead to faster emotion discrimination in HCs (42), which is in line with the strengthened response to (negative) images in HCs after rTMS in our study. Together, these studies imply that high-frequency right dlPFC rTMS impacts emotion processing in HCs, but the neural mechanisms through which these effects occur may partly depend on the paradigm used, which differ between these studies, and are thus in need of further study.

These results are not in line with a recently published multilevel framework on explicit and implicit emotion regulation (18), since the dlPFC is associated with explicitly controlled emotion regulation whereas no effect of dlPFC stimulation on reappraisal was found within this study. The effects on emotion processing reported here may have been caused by (subthreshold) activity changes beyond the site of stimulation that have previously been reported in rTMS studies (66), although none of these effects were found in the fMRI analyses. It is possible that other stimulation targets will render different results; the dmPFC and insula have, for example, been suggested as alternative targets for rTMS stimulation treatment in substance use disorder (67).

Although expected, we did not find any effect of rTMS on experienced emotion, or related brain activity in response to alcohol-related images. This may be explained by the variation in image content, individual preferences for certain alcohol-related contexts, or specific beverage preferences. The images used in the emotion reappraisal task consisted of different variations of alcoholic beverages (beer, wine, and liquor) and alcohol-related contexts (e.g., bar and supermarket). Alcohol-related images may elicit different (e.g., positive and/or negative emotional) responses in AUD patients specifically, due to the psychological burden of having an AUD, and it is possible that these individual differences thus did not result in consistent emotional and brain responses in the AUD group. Increasing the sample size or selecting a subsample of, e.g., beer- or wine-preferring AUD patients, in order to analyze subgroups with specific preferences could clarify these results in future studies.

Finally, in our previous study within this special issue (68), we show that the emotion reappraisal task increases craving levels in both AUD patients and HCs, and that AUD patients have higher overall craving levels. In the current study, we show not only that rTMS affects craving levels differently in AUD patients and HCs but also that the time by stimulation interaction was not significant. These results do not support our hypothesis that rTMS reduces craving levels compared to sham stimulation and are not in line with our meta-analysis on this topic (31), but suggest an accidental preexisting difference in craving levels between the stimulation groups. Furthermore, AUD patients were not eligible for participation when actively using psychoactive medication, including anti-craving medication, due to possible confounding effects on the fMRI data. However, inclusion of non-medicated AUD patients may have resulted in a selection bias. Possibly, these nonmedicated patients are (compared to medicated patient samples) less prone to craving and less susceptible to induction of craving by the emotion reappraisal task. Also, recent reviews (29, 30) suggest that neurostimulation techniques may be more effective in reducing craving for substance use disorder patients when applying more (and longer) stimulation sessions. Finally, although the current study included a larger sample compared to previous neurostimulation and fMRI studies on emotion processing and reappraisal, the sample is still modest, requiring larger effect sizes (or more neurostimulation sessions) to obtain significant results. Future studies should therefore apply more stimulation sessions in a larger AUD sample in order to establish if the rTMS effects reported in this paper are clinically relevant.

### Conclusion and Future Directions

This study is the first study that indicates differential effects of sham and high-frequency right dlPFC rTMS on emotion processing, reappraisal ability, and related brain functions in AUD patients and HCs. Subjective experienced emotion during the emotion reappraisal task was reduced after right dlPFC rTMS in AUD patients, but increased the subjective experience in HCs. This possibly indicates an rTMS-related impact on emotion processing of emotional (but not alcohol-related) images in AUD patients. rTMS stimulation changed brain activity in various emotion reappraisal relevant brain areas but did not reduce craving levels in AUD patients. Future studies should replicate and expand the current study, for instance, by assessing the effect of multiple stimulation sessions on both explicit and implicit emotion regulation paradigms and craving, and assess the effect of rTMS within subgroups with specific addiction-relevant image preferences.

## Ethics Statement

This study was carried out in accordance with the recommendations of medical ethical committee of the University of Amsterdam Medical Centre, with written informed consent from all subjects. All subjects gave written informed consent in accordance with the Declaration of Helsinki. The protocol was approved by the medical ethical committee of the University of Amsterdam Medical Centre.

## Author Contributions

All authors made a significant contribution to this article, including acquiring funding (AG, WB), study design (AG, WB), development of the emotion reappraisal task (SW, OH, DV, YD), data acquisition (JJ), data analysis (JJ, DV, SW), interpretation of results (JJ, AG, SW, OH, YD, WB, DV), and contributions to this manuscript (JJ, AG, SW, OH, YD, WB, DV).

## Funding

This research was partly funded by The European Foundation for Alcohol Research (ERAB), grant no. EA1027 to AG, WB, and DV and by a VIDI grant (no: 91713354) from the Dutch Scientific Foundation to AG.

## Conflict of Interest Statement

The authors declare that the research was conducted in the absence of any commercial or financial relationships that could be construed as a potential conflict of interest.
