# Supplementary material for: The Effect of High-Frequency Repetitive Transcranial Magnetic Stimulation on Emotion Processing, Reappraisal, and Craving in Alcohol Use Disorder Patients and Healthy Controls: A Functional Magnetic Resonance Imaging Study
Source: Front Psychiatry. 2019 May 7;10:272. doi: 10.3389/fpsyt.2019.00272 (PMC6516054; doi:10.3389/fpsyt.2019.00272)
Supplement: Supplementary file 1 [file Data_Sheet_1.docx]

Supplementary Information

**Supplement 1**

| Brain Area | L/R | F | K | X | Y | Z | P-value (FWE-corrected) |
| --- | --- | --- | --- | --- | --- | --- | --- |
| rTMS effect: ADP > HC |  |  |  |  |  |  |  |
| dlPFC | right | 25.89 | 80 | 24 | 29 | 34 | .019 |
| rTMS effect: HC > ADP |  |  |  |  |  |  |  |
| Supramarginal gyrus | left | 24.57 | 49 | -30 | -37 | 31 | .032 |

Supplementary figure 1. Emotion processing. This figure shows the interaction between participant group (ADP, HC), and stimulation (rTMS, sham).

| Brain Area | L/R | F | K | X | Y | Z | P-value (FWE-corrected) |
| --- | --- | --- | --- | --- | --- | --- | --- |
| rTMS effect: |  |  |  |  |  |  |  |
| Superior frontal gyrus | right | 10.01 | 47 | 21 | 5 | 52 | .07 |
| Superior frontal gyrus | left | 9.98 | 13 | -21 | 5 | 49 | .07 |

Supplementary figure 2. Emotion reappraisal. This figure shows the interaction between emotion (alcohol, neutral, positive, negative) and stimulation (rTMS, sham).
